# Challenges and Opportunities for Printed Electrical Gas Sensors

**DOI:** 10.1021/acssensors.2c01086

**Published:** 2022-09-22

**Authors:** Giandrin Barandun, Laura Gonzalez-Macia, Hong Seok Lee, Can Dincer, Firat Güder

**Affiliations:** † Imperial College London, Department of Bioengineering, Royal School of Mines, SW7 2AZ London, United Kingdom; ‡ BlakBear, Ltd, 7-8 Child’s Place, SW5 9RX London, United Kingdom; § FIT Freiburg Center for Interactive Materials and Bioinspired Technologies, University of Freiburg, Freiburg 79110, Germany; ∥ Department of Microsystems Engineering (IMTEK), University of Freiburg, Freiburg 79110, Germany

**Keywords:** gas sensing materials, sensing technology, gas sensor applications, printed gas sensors, health monitoring, air pollution monitoring, food freshness sensing

## Abstract

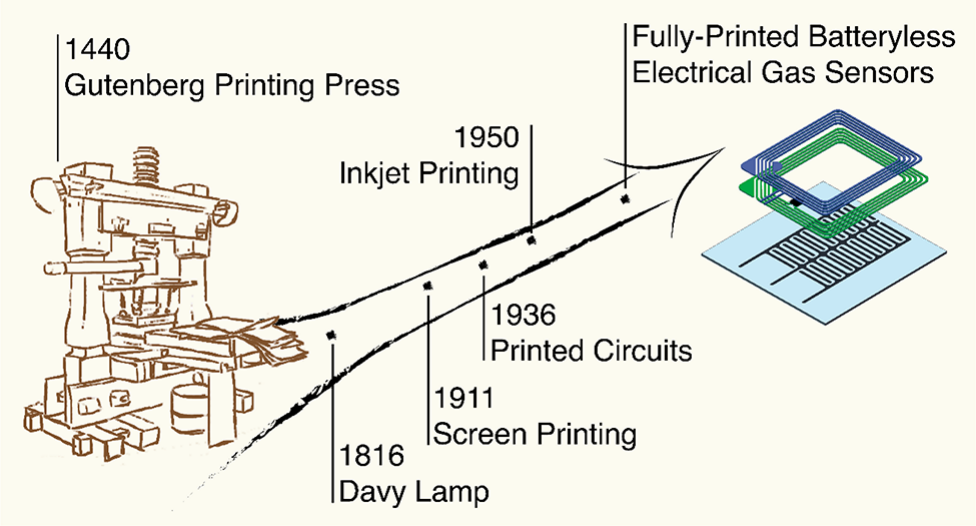

Printed electrical gas sensors are a low-cost, lightweight, low-power, and potentially disposable alternative to gas sensors manufactured using conventional methods such as photolithography, etching, and chemical vapor deposition. The growing interest in Internet-of-Things, smart homes, wearable devices, and point-of-need sensors has been the main driver fueling the development of new classes of printed electrical gas sensors. In this Perspective, we provide an insight into the current research related to printed electrical gas sensors including materials, methods of fabrication, and applications in monitoring food quality, air quality, diagnosis of diseases, and detection of hazardous gases. We further describe the challenges and future opportunities for this emerging technology.

Printing is an ancient idea that has been reinvented in different forms countless times over several millennia (Figure 1).^1^ Printing aims to massively parallelize or automate serial manufacturing for reproduction of text, images, and physical objects to reduce cost, time, and materials. Printing was initially used for producing art, spreading ideas, and storing information; therefore, in addition to being a manufacturing method, printing is a communication technology much like the telephone. Printing has also played a pivotal role in the electronics revolution that enabled low-cost, miniaturized, and highly integrated analog and digital electronics with the introduction of printed circuit boards (PCBs).^2^ Although PCBs are currently produced using subtractive manufacturing (that is, by chemically etching traces of Cu), new electronic inks that could be inkjet, screen, or stencil printed are increasingly being used for the additive manufacturing of printed electronics and sensors.^3^ Up until the 1980s, however, printing was mainly used for producing planar structures–text, images, or thin layers of material were solely deposited on a flat surface. With the invention of 3D printing (another additive manufacturing method), layers were deposited on one another guided by a computer, allowing fabrication of complex device geometries with high fidelity that could not be easily produced with other methods.^4^

**Figure 1:**
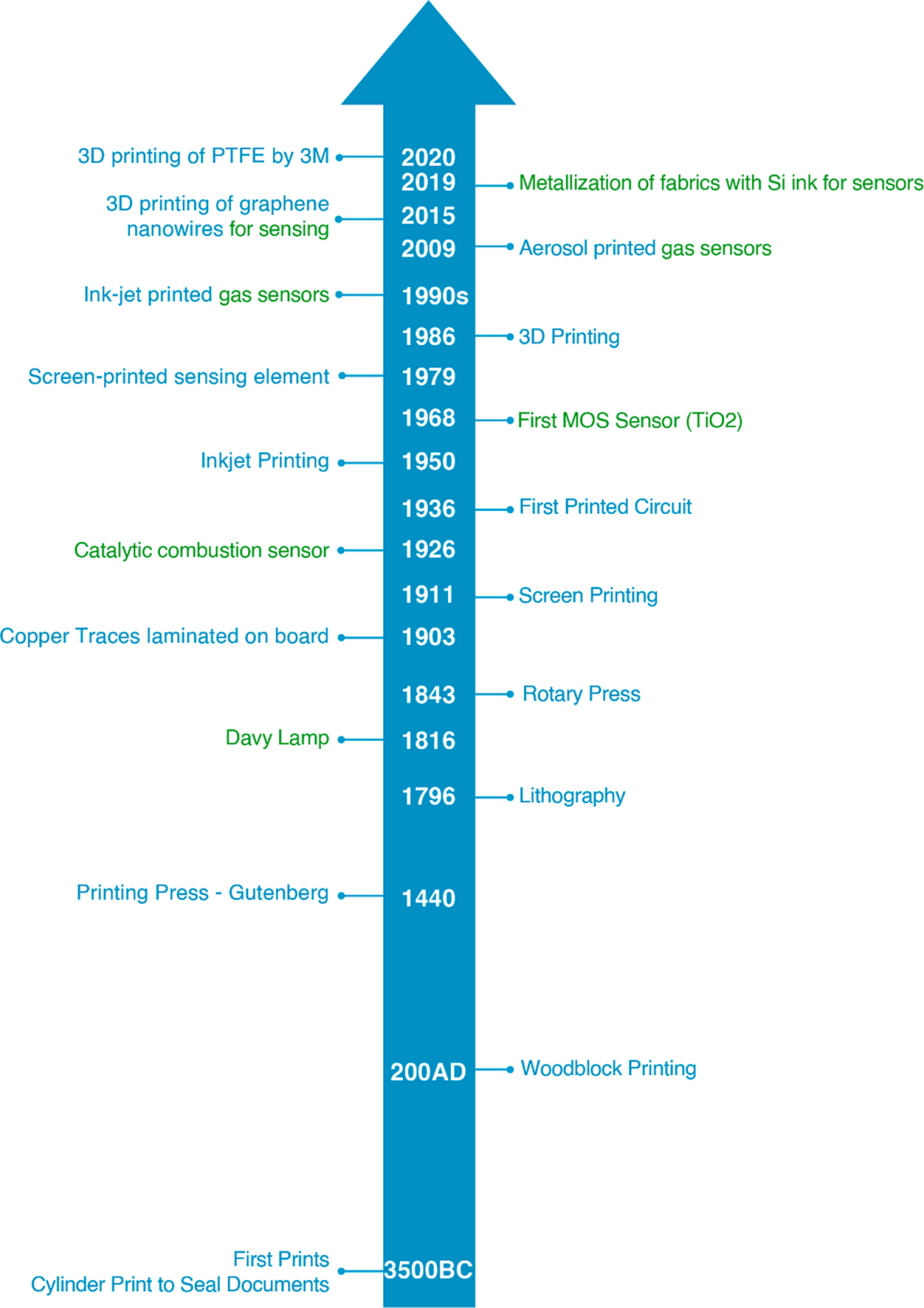
Brief timeline of important events in printing (blue) and gas sensing (green) technologies.

While the history of printing stretches back several millennia, the era of gas sensors started in 1816 with the Davy lamp.^5^ One hundred years later the first electrical gas detector was invented by Oliver Johnson for measuring combustible gases in the atmosphere.^6^ In the early 1960s, Naoyoshi Taguchi developed the first commercial electrical gas sensor due to the increasing propane gas explosions in Japan.^7^ The metal oxide semiconductor (MOS) gas sensor developed by Taguchi had a simple architecture and consisted of a tin oxide layer deposited on a ceramic substrate. The electrical resistance of the tin oxide layer changed upon exposure to various gases. Later, Taguchi founded Figaro, a company which is still operating today.^8,9^ Starting in the 1970s, research into gas sensors increased, accelerating more in the early 2000s.^10^ Because of the increased activity in the field of sensing, the first decade of the 21^st^ century was named the “sensor decade”.^11^ Over the past few years, a merging of printing techniques and gas sensing technologies has begun. Significant efforts have been made to adapt existing printing techniques to the fabrication of gas sensors, leading to many successful examples of electrical and colorimetric gas sensors. A colorimetric gas sensor changes color when exposed to a gaseous analyte and is often read with the naked eye. A camera or, in simple configurations, a single photodiode can also be used to perform readings. The use of additional instruments to perform readings, however, increases complexity and cost. In contrast, electrical gas sensors convert chemical and/or physical changes into electrical changes (for example, resistance or capacitance) when exposed to a target gas. These electrical changes can be registered as electrical signals such as variations of voltage or current. Due to the simplicity of the mechanism of transduction, most printed electrical gas sensors are chemiresistive^12,13^ or (more elaborate) field-effect transistors (FET).^14,15^ Chemiresistive and FET type devices typically consist of a thin layer of sensing material deposited between two electrodes, the conductance of which changes in the presence of a target gas.^16^ Other types of electrical gas sensors include electrochemical sensors^17,18^ and diodes.^19,20^ These two types are less common in printed conformation due to the requirements of high feature resolution or additional modification. Electrical gas sensors are considered to be higher performance sensors in comparison to their colorimetric counterparts (see Table 1 for the typical analytical metrics for assessing the performance of a gas sensor). The signals obtained from electrical gas sensors are easier to interpret as they produce an absolute electrical value instead of a relative color change that is often subjective and affected by ambient lighting. Electrical gas sensors are also easier to integrate into mixed-signal electronic systems and Internet-of-Things networks. Hence, they are compatible with existing and future connected solutions.

**Table 1:**
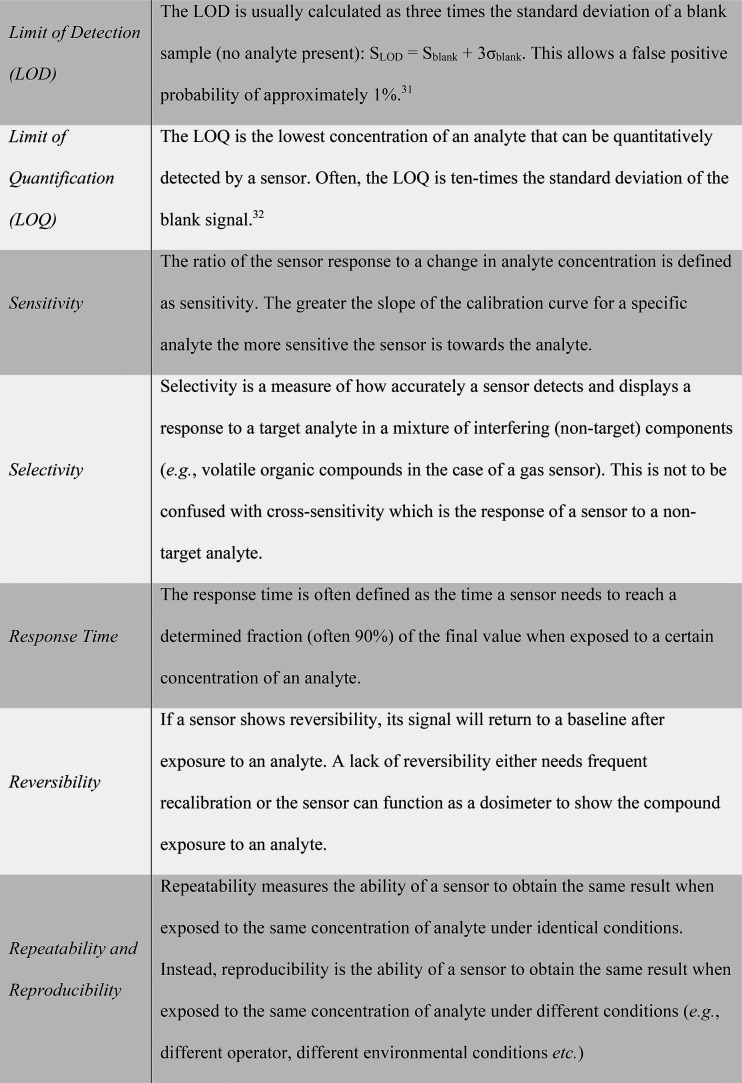
Typical Analytical Characteristics of Gas Sensors

Screen printing was the first printing technique used for the fabrication of printed electrical gas sensors, where a semiconductor paste was used to print the sensing element.^21,22^ Inkjet printing was later used for the fabrication of electrical components and gas sensors using conductive polymer-based inks.^23−28^ Although screen printing and inkjet printing are probably the most popular methods for fabricating electrical gas sensors, techniques such as aerosol printing or 3D printing have also been used.^29−32^ The term “printed electrical gas sensor” is often used when referring to the sensor unit (consisting of the gas-sensitive material and electrodes) and not the entire device, often leading to confusion. The sensor unit is a part of a fully integrated sensor system, responsible for the detection of the target gas and its transduction into quantifiable electrical signals. The signals originating from the sensor unit are converted to easily measurable electrical signals (for example, converting resistance change into voltage) using electronics before digitization and subsequent transmission to a read-out instrument (for example, a display on the same system, a nearby smartphone or computer). Electrical sensor systems can be powered actively with a printed battery or passively by wireless power transfer (WPT) involving transmission of power from a reader, such as a smartphone, wirelessly. Typical elements of a printed electrical gas sensor system are summarized in Figure 2.

**Figure 2:**
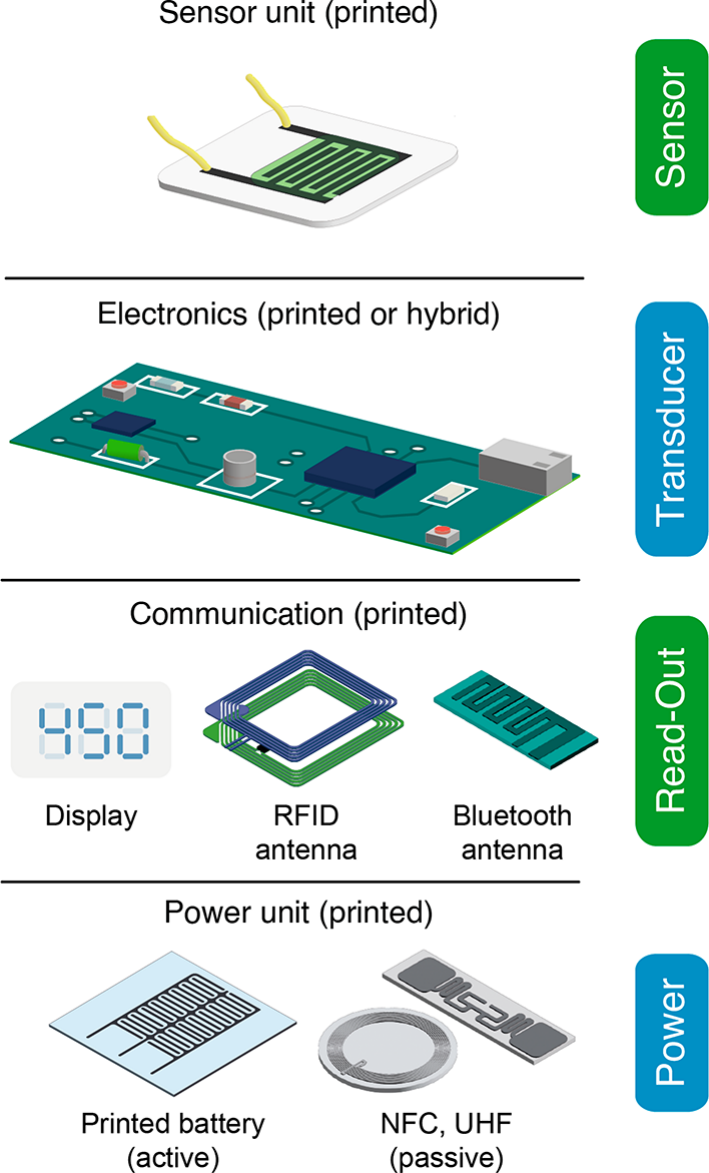
Elements of (printed) gas sensing systems.

In this perspective, we will describe the latest advances in printed electrical gas sensors and the challenges that will need to be overcome to realize integrated printed gas sensing systems. We will provide insights into current and future applications and give examples of the use cases for printed electrical gas sensor systems.

## Fabrication of Printed Gas Sensors

To fabricate printed gas sensors, three elements would need to be considered depending on the application: (i) the *Substrate* is the carrier on which the functional materials, electrodes/interconnects, and other components are deposited; (ii) *Inks* are the materials required to print the sensing unit, interconnects, and other functional elements such as heaters and membranes to operate the sensor or improve its performance; and (iii) the *Printing method* defines the scale of manufacturing in addition to the inks and substrates that can be used in fabrication. Hence, all three elements that would need to be considered for the fabrication of printed gas sensors are interdependent. For example, the substrate will restrict the type of materials to be deposited and the printing method to be used during fabrication.

## Substrates

There is a wide range of materials that can be used as substrates for the fabrication of printed electrical gas sensors, including organic and inorganic materials. While organic substrates can be flexible or rigid depending on the chemistry and formulation used, inorganic materials, such as ceramics, are primarily used as rigid substrates.^33,34^ Though low-cost and compatible with various large-volume production methods such as roll-to-roll printing, organic substrates are not compatible with fabrication and operating sensing regimes that require high temperatures. For example, polyethylene terephthalate (PET), a common substrate used in the fabrication of printed devices, has a glass transition temperature of below 150 °C whereas many inks employed in screen and inkjet printing need higher temperature for the curing process.^35,36^ Additionally, numerous gas sensors require high temperatures (>200 °C) to operate due to the inherent nature of their sensing materials (for example, metal oxides)^37,38^ or their application (for example, automotive industry, agriculture waste processes, nuclear power plants, aerospace industry).^39,40^ Inorganic substrates such as ceramics^41−43^ and silicon derivatives^44,45^ have been traditionally used because of their compatibility with high temperatures and resistance to harsh environments. With the rise of wearables and smart packaging labels, however, there is increasing interest in the use of flexible and stretchable substrates for the fabrication of printed electrical gas sensors.^46−48^ Extensive research is currently dedicated to improve thermal properties of flexible materials and to lower curing and operation temperatures of printable inks to enable the integration of printed gas sensors into those applications.^49−52^ Because of potential contact with skin and food, biocompatibility and toxicity have become important criteria in addition to the mechanical properties of the substrates.^47^ The most common flexible substrates used for the fabrication of gas sensors apart from PET are polyethylene-2,6-naphthalate (PEN), polydimethylsiloxane (PDMS), and polyimide (PI).^53,54^ They are preferred to foils of metal because of their robustness, low cost, and (insulating) electrical properties. More recently, porous 3D organic materials such as paper and textiles have emerged as alternatives to planar polymer films as substrates for printed gas sensors due to their low cost, flexibility, improved gas exchange, compatibility for mass production, and availability in well-established industries.^55−57^ Inks printed on porous substrates penetrate the substrate, allowing 3D fabrication of robust, printed electrical gas sensors.^10,58^

## Inks for Printed Gas Sensors

**Figure 3:**
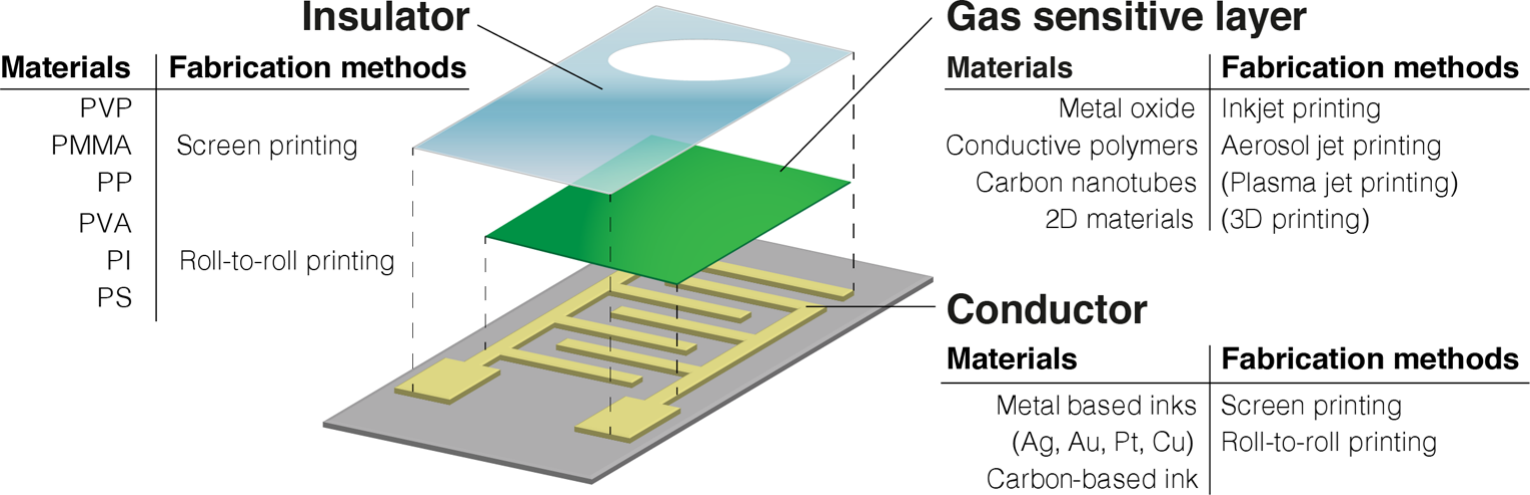
Individual components of a gas sensor comprising electrodes, gas sensitive film, and an insulator layer.

Inks for the fabrication of printed electrical gas sensors typically comprise two or more of the following four components depending on the printing method used: (i) functional materials such as metallic or semiconducting nanomaterials, conductive polymers, 2D nanostructured materials, or carbon-derived materials to act as gas sensitive materials or to construct electrodes/interconnects;^16,59−63^ (ii) binders such as glass powder, resins, or cellulose acetate to hold together functional particles and provide adhesion to the substrates;^64−66^ (iii) solvents such as water, ethylene glycol, terpineol, or cyclohexanone to enable printability; and (iv) other additives such as wetting agents for inkjet printing as stabilizers.^16,67^ The presence, type, and quantities of each component will define the rheological properties of the ink according to the requirements of the printing method. For example, inks intended for inkjet printing require low viscosities (4–30 mPa·s) to enable the formation and ejection of droplets from the nozzle(s) (<100 μm diameter) and high surface tension (20–70 dyn cm^–1^) to avoid dripping during the process. Inks for more conventional printing require higher viscosities (100–2k mPa·s for gravure and flexographic, and up to 10k mPa·s for screen printing) and more restricted surface tensions (∼40 dyn cm^–1^ for gravure, 28–38 dyn cm^–1^ for flexo or 30–50 for screen printing) to avoid leakage during transfer to the substrate. High viscosities are normally achieved by increasing the percentage of binder or decreasing the solvent ratio in the ink formulation.^64,66,67^ The surface tension of the inks is primarily defined by the solvent used: water-based inks possess high surface tension (water surface tension is 73 mN m^–1^) whereas the surface tension of nonpolar solvents is generally low. Surface tension can be reduced by adding low molecular weight alcohols and surfactants or increasing the particle concentration in the inks, which improves substrate wetting although it can affect the viscosity. Surface treatment of the substrates (for example, by oxygen plasma or ozone) can improve the deposition of the inks without affecting the ink formulation.^66,68^ Inks are formulated according to the functional material, substrate, and preferred printing method, whereas the rest of ink components (binders, solvents and additives) are just the medium to enable the material deposition. The preparation of the ink generally starts with the development of the varnish (binders, solvents, and additives) followed by the addition of the functional materials assisted by dispersion technologies (such as ball milling). Final adjustments in the formulation are then performed to fulfill the rheological properties.^64^ Inks used in the fabrication of printed electrical gas sensors can be classified into three main groups (Figure 3):

i. *Inks for printing gas sensitive materials*. The gas sensitive material defines the majority of the sensing properties of the printed electrical gas sensor, including sensitivity and selectivity for the target analyte, response time, reversibility, and stability.^69^ Inks consisting of gas sensitive materials such as metal oxide particles (SnO_2_, CuO, In_2_O_3_, WO_3_),^34,61,70,71^ conductive polymers (polyaniline, poly(3,4-ethylenedioxythiophene):polystyrenesulfonate, polypyrrole),^72−74^ carbon nanotubes (CNTs),^60^ 2D materials (such as graphene),^75,76^ and more recently, combinations of these have been reported.^77−79^ Combining different gas sensitive materials, such as conductive polymers and nanomaterials, often lead to enhanced ink processability and sensing performance.^80,81^
ii. *Inks for printing conductors*. Conductive inks are primarily made of metallic particles such as Ag, Cu, Au, or Pt because of their high conductivity in comparison to carbon-based materials. Ag is the most preferred metal filler due to its stability against oxidation and reasonable price compared to other noble metals.^82,83^ Ag-based inks are used for the fabrication of interconnects, coils, and antennas by gravure, inkjet, or screen printing.^84,85^ Wirelessly powered gas sensor systems (for detecting H_2_S, O_2_, CO_2_, NH_3_) with printed Ag antennas have already been reported in the literature.^86,87^ Bimetallic nanoparticles comprising a core of highly conductive, low-cost metal, such as Cu or Ni, and a protective shell of Au or Ag, are emerging as an alternative to noble metal nanoparticles to reduce the cost of inks.^88^
iii. *Inks for printing dielectrics*. Standard (liquid-phase) electrochemical printed sensors normally include dielectric insulator films to define the surface area of the sensors and shield the electrical contacts from the solution to prevent short-circuiting between electrodes and artifacts in the output signal. For gas sensors, dielectric membranes based on PI, polyvinylphenol (PVP), poly(methyl methacrylate) (PMMA), polypropylene (PP), poly(vinyl alcohol) (PVA), and polystyrene (PS) can also be part of the transducer system to construct capacitors or thin-film-transistor-based sensors.^89,90^ These materials are also used as support for gas sensitive composite inks and as selective membranes for gas sensors.^91−93^

In addition to the classes of inks described above, other organic polymers with electro-optical properties such as electroluminescence (EL) and electrochromic (EC) characteristics are currently of high interest for printed displays and (organic) light emitting diodes (OLEDs).^94−96^ The combination of multiple printing methods to deposit materials of various nature and rheological properties has also enabled the printing of components such as batteries and photovoltaic modules, promising for flexible sensing applications.^90,97^

## Printing Methods

There is a large range of printing technologies available today for creating electrical gas sensors: screen, inkjet, roll-to-roll (such as gravure, flexographic, and nanoimprint/hot embossing), 3D, aerosol and plasma jet, stencil, and transfer printing.^16,53,77,98−101^ We only briefly introduce each method here to base the discussion on printed gas sensors – a comprehensive description of each technique and its operation can be found in general reviews on printing methods.^64,66,67^

Screen printing is the most mature and widely used printing method. The ink is transferred to the substrate through a stencil screen by applying pressure using a rubber squeegee. Screens are normally made of a mesh of fabric, synthetic fibers, or metal threads and contain a negative image of the required pattern deposited by photolithography.^102^ Screen printing is amenable for planar (sheet-by-sheet) or roll-to-roll configuration.

During gravure printing the pattern engraved into a rotatory cylinder is inked and transferred to a substrate by bringing them into contact. Flexography relies on a similar mechanism, but the pattern is mounted on a second cylinder with flexible printing plates. The ink is collected by the first cylinder and transferred to the second cylinder, which deposits the ink onto the substrate. Like gravure and flexography, roll-to-roll nanoimprint/hot embossing facilitates the transfer of microstructure patterns from the cylinder mold onto the substrate by pressure. Additional heating is required to reach temperatures above the glass transition temperature of the polymer during the patterning process.

Roll-to-roll techniques can reach printing speeds of up to 1000 m/min, which require inks with low boiling points solvents to accelerate the drying process. Resolutions are in the order of 100 μm, although recent research has allowed to push the limits down to the tenths of micrometers.^103^

Inkjet printing enables the highly controlled deposition of low volumes of ink (picolitres) onto a substrate with high precision and reproducibility. The ink is ejected from a nozzle in a continuous or drop-on-demand mode – the most common inkjet printers are based on drop-on-demand piezoelectric or thermal mechanisms.

Less conventional, 3D printing has recently emerged as a promising solution for the manufacture of nonplanar structures. Typical inks are plastic filaments (polycarbonate (PC), acrylonitrile butadiene styrene (ABS) and polylactic acid (PLA)) extruded at the printhead enabling the layer-by-layer construction of the design onto the substrate. Extensive research is ongoing to achieve the deposition of functional materials by other variations of 3D printing, extending the use of this technique further than device housing cases and scaffoldings.^104−106^

During aerosol printing, an aerosol of ink microdroplets is created inside the atomizer and transported to the printhead by a carrier N_2_ flow. This technique enables resolutions of 10 μm, lower than inkjet and screen printing, and has facilitated the recent fabrication of all-aerosol-jet-printed gas sensors for NH_3_.^99^

Stencil printing can be considered a similar but simpler version of screen printing. The ink is transferred through a mask to a substrate to create the required pattern. The mask contains a hollow image of the design, which complicates the control of layer thickness and resolution. Stencil printing is normally applied to connect the components of the electrical circuits rather than the sensing unit.^107^

Transfer printing resembles flexography and microcontact printing – a soft stamp (normally made of PDMS) transfers microstructures previously patterned on one substrate (donor) to another substrate (receiver) based on differences between material affinities. It is an emerging technology for flexible and stretchable electronics manufacturing, where pattern thickness of conductive materials as small as a few micrometers have recently been achieved.^108,109^

Conventional contact methods (like screen and roll-to-roll) enable high-quality manufacturing of predesigned hard patterns (through screens or cylinders) whereas more recent noncontact methods (like inkjet, aerosol, and 3D) use digital designs. The printing method used when fabricating a device depends on the minimum feature size, type of substrate, availability, and cost of the inks and their function (for example, sensing material, conductor, insulator). For example, screen printing is widely used to deposit electrodes, interconnects, and insulators, which do not require high resolution (>100 μm); a wide range of inks are also available in the market. The sensing material, however, is typically deposited by inkjet printing.^83,100^ Inkjet printing, though versatile, is not suitable for mass manufacturing, and it is therefore mainly used for prototyping. Roll-to-roll methods, however, require large quantities of ink (>1 L), which is not compatible with the early stages of printed sensor development.^64^ Because of their speed, cost, and simplicity, screen and roll-to-roll printing techniques are the preferred method for mass production of gas sensors at a mature stage.^16,67^

Gas sensors are already being manufactured using one or a combination of the above-mentioned printing techniques. More complex electronic components requiring high patterning resolution (<10 μm) are, however, still fabricated using traditional processes such as photolithography, spin-coating and etching.^89^ Control of thickness and pattern resolution are two of the main challenges for printing techniques, although many advances have been made to date, including the use of alternative sintering methods to cure printed conductive inks.^16,85,90,110^

## Device Integration

Although it is certainly possible to fabricate electrical gas sensors and interconnects, creating a fully integrated gas sensor system by printing is still an unsolved problem; new printing technologies and materials are needed to replace silicon-based electronics for computational and analog operations.^110^ For example, in a recent article Lin et al. fabricated a self-powered gas sensor, where an amorphous silicon (a-Si) solar cell array converted light into electricity to power a SnO_2_-based gas sensor for measuring vapors of acetone and ethanol. The device, which included a supercapacitor, was inkjet-printed on a flexible PET substrate to create a wearable wristband sensor system. The fabrication of the gas sensing, power storage, and supply units on a single device via printing is a step toward realizing all-printed gas sensors, although, some of the electronic components (for example, voltage regulator and surface mounted LED as a warning display) were not printed.^54^

Because creating fully integrated electrical gas sensing systems by printing is currently not feasible, commercial solutions integrate components produced using a range of manufacturing technologies. For example, Spyras’ Smart Facemask combines paper-based printed humidity sensors with conventional electronics and data analysis to track respiratory patterns.^111^ SPEC sensors combine screen-printed electrochemical sensors with conventional electronics for monitoring air quality (NO_2_, CO, SO_2_, H_2_S) and breath ethanol.^112^ Altered Carbon Ltd. provides customized graphene ink to print sensor arrays for a variety of gases.^113^ BlakBear Ltd. integrates paper-based electrical gas sensors with wirelessly powered conventional electronics to provide smart labels for the monitoring of food spoilage.^114^ BreathDX AmBeR offers disposable printed sensors in combination with an external analyzer for the quantification of ammonia in-breath.^115^ Scalability will be enabled in the next few years (passing through hybrid systems first) by the adaptation of the gas sensing technologies to other areas such as wearables. Further understanding of material science, fluid mechanics, and printing techniques is still crucial to fulfill integration of printed gas sensors and to assist the translation of lab prototypes into commercial products.

## Applications

In this Perspective, we primarily attempt to link printed electrical gas sensors to applications in food spoilage, air quality, health monitoring and detection of hazardous gases. Sensors for ammonia (and its derivatives such as methylamine and trimethylamine)^28,116−121^ nitrogen dioxide (NO_2_)^122−125^ and volatile organic compounds (VOCs)^126−129^ are the most studied gas sensors in the literature. NH_3_ is an important indicator of food spoilage in protein-rich foods^130^ and an indicator of health when measured in exhaled breath.^131^ The human sensory threshold for NH_3_ is ∼50 ppm, a relatively low detection threshold (though heavily dependent on the individual).^132^ For some applications, however, dedicated sensors are still necessary, such as air quality monitoring (<1 ppm) or exhaled breath analysis (0–10 ppm). The limit of concentration where NH_3_ poses an immediate danger to health is at 300 ppm, well above the sensory threshold of humans;^133^ in contrast, NO_2_, an important air pollutant, can present an immediate danger to life and health at concentrations as little as 12 ppm^134^ and accepted concentrations in the atmosphere in the U.S. are 0.053 ppm as an annual mean and 0.100 ppm as an hourly mean.^135^ VOCs, however, are relevant as analytical targets for monitoring air quality, measuring food quality, and assessing human health.

## Food Spoilage and Freshness

Printed sensors can be used to detect the freshness of protein-rich products (for example, NH_3_ for fish and meat), ripeness of fruits (ethylene), and integrity of packaging in packaged foods with modified atmospheres (CO_2_, O_2_).^136^ Although there is a vast number of reports in the literature concerning smart packaging, gas sensors for measuring food spoilage and quality,^137−141^ many of the works published have major shortcomings. The sensors reported: (i) are not cost efficient enough to be implemented in (disposable) food packaging; (ii) use toxic materials that are not suitable for food contact; (iii) require high power for operation; (iv) are not stable long-term under packaging conditions (high relative humidity (RH)); and (v) lack sensitivity.

With current technologies, it is difficult to gather real-time data on the biological state of food products across the supply chain. For example, the freshness of raw poultry at any given time is often debated and no agreed metric exists. The subjective olfactory threshold of humans for vaporous bacterial metabolites is often used as a measure for identifying spoilage (for example, for poultry or fish). Using this metric, the shelf life of raw chicken is limited by the subjective detection of metabolites (mostly sulfuric metabolites, for example, hydrogen sulfide (H_2_S)).^142−144^ An integrated printed gas sensor in packaged poultry can give real-time insights on presence and concentration of volatile metabolites. Such an implementation in raw poultry packaging will need to fulfill the following three requirements: (i) *Cost*. The printed sensor system must not increase the total cost of packaging substantially (U.S. ¢1); (ii) *Long-term operational stability under packaging conditions*. The average shelf life for raw chicken is 10 days from kill date. When most foods are packaged, the atmosphere inside the package can be altered to increase its lifetime. For example, the amount of oxygen can be reduced to limit the growth of aerobic bacteria or vacuum can be created to additionally limit the growth of anaerobic bacteria. For raw chicken, often *m*odified *a*tmosphere is used for *p*ackaging (MAP, for example, 80% N_2_, 20% CO_2_). The printed sensor must remain chemically and electronically stable within the packaging; (iii) *Integration*. The printed sensor system needs to be easily integrated into the existing packaging processes.

There are several notable reports that try to produce low-cost printed sensors for measuring food quality. Koskela et al. inkjet printed copper acetate (CuAc) on paper and PET to produce a H_2_S sensor.^145^ They used inkjet or roll-to-roll printed silver to form the electrodes to the sensing layer. In the presence of H_2_S, CuAc forms copper sulfide, irreversibly modifying its electrical resistance. They evaluated the printed sensor at different packaging atmospheres and temperatures (Air, MAP, room temperature (RT), 6 °C); The sensor printed on the paper substrate showed a dependency on RH above 80%. This effect occurred because paper, a hygroscopic material, adsorbs moisture from its immediate environment which, in this case, parasitically impacted the conductivity of the printed sensor. The PET substrate, however, showed a negligible dependence on RH, improving H_2_S detection. The sensors produced were subsequently integrated into an RLC circuit for wireless measurements. The printed sensors could be probed with an electronic article surveillance (EAS) reader to measure the quality factor (*Q*) which changed when the resistance of the printed sensor changed due to reaction with H_2_S. This wireless sensor system has not yet been tested with spoiling meats.

Total volatile basic nitrogen (TVBN), consisting of NH_3_, methylamine, trimethylamine, and the larger molecules cadaverine and putrescine, are also used in assessing the freshness of food products.^146−148^ The concentration of these gases can range from a few ppm in the early days (<5 days) to hundreds of ppm at end-of-life of meats. Printed ammonia sensors have been produced using a variety of printing technologies including inkjet,^80,149,150^ aerosol jet,^151^ and 3D printing,^152^ using carbon-based,^150,151^ conductive polymers,^80,153^ or semiconducting materials.^152^

Barandun and co-workers developed a printed low-cost impedimetric gas sensor (carbon ink on cellulose paper) with a lower limit of detection (LOD) of 0.100 ppm for NH_3_ and also exhibited sensitivity toward other TVBN.^55^ The conductance of the sensor increased up to 1000% when placed in a sealed environment containing a fillet of cod over 10 days in a household fridge. Additionally, the paper-based printed gas sensor was combined with a commercially available Near-Field Communication (NFC) tag to produce an on/off type wireless spoilage sensor: when the resistance of the sensor dropped under a concentration threshold of a target gas, the tag stopped responding to the reader (for example, an NFC-capable smartphone) since the printed sensor shunted the NFC chip, diverting the electrical power to the sensor and not the chip. Although easier to implement than quantitative sensors, on/off (binary output) type sensors are not useful for food supply chain monitoring and for predicting use-by dates. Binary output sensors can, however, be useful for indicating spoilage and reduce the risk of food poisoning or consumer complaints. BlakBear Ltd.^114^ is currently advancing this technology for measuring the spoilage of protein-rich food products by developing a passively powered, quantitative, printed, ultrahigh frequency (UHF) wireless label for integration into food packaging. In contrast to the range of a few centimeters for NFC, UHF can supply power and read tags over a few meters, enabling continuous measurement from a distance.

In another study Ma et al. presented a wireless printed sensor system with inkjet-printed nanostructured polyaniline (PANI) printed over the NFC tag antenna.^154^ The PANI had a low initial resistance and shorted the antenna traces which rendered the tag unfunctional. Upon exposure to amines, the resistance of PANI increased, making the tag functional at a certain threshold. The lowest tested concentration was 5 ppm of ammonia, which can give an indication of increased ammonia levels in later stages of the shelf life of meat products (>5 days).

In fruits, ethylene is used as an indicator for ripeness.^155^ Detecting ethylene is more challenging than ammonia. Hence, ethylene is often measured by gas chromatography, optical sensors, or electronic noses (e-noses).^156−158^ These systems are expensive and complex. Although not fully printed, there have been a few reports of chemiresistive sensors to measure ethylene, but more research is needed to produce low-cost and fully printed sensors.^159,160^

If food packages are not sealed perfectly, leakage can occur and over the course of the lifetime of the product, the quality can drop drastically. To detect leaks in packaging, CO_2_ sensors can be used. Andó et al. described a fully printed CO_2_ sensor.^161,162^ They applied PEDOT:PSS and graphene ink, using a spreader, onto inkjet-printed silver electrodes. To operate the sensor, however, consecutive heating cycles were required, achieved by placing a heater under the sensor. This made the entire system more complicated, power hungry, and difficult for integration into food packaging. Power consumption, in general, is a key factor that determines the viability of integration of printed electrical gas sensors into low-cost packaging.

## Environment

Air pollution is one of the biggest challenges in environmental monitoring and can cause a variety of diseases including asthma, heart diseases, cancer or pulmonary illnesses.^163^ It is considered to be the largest environmental health threat with 7 million deaths each year, yet proper monitoring infrastructure is often missing.^164^ Air pollution is affecting 92% of the world’s population, with up to 98% of children breathing toxic air in developing countries.^165^ Conventional air pollution monitoring is performed with large, expensive monitoring stations which are sparsely distributed, leading to low spatial resolution.^166^ A large network of low-cost, low-power, printed air pollution sensors could provide a high resolution map of air quality and lead to creation of scientifically backed policies to improve air quality in major population centers.

One of the most important requirements for gas sensors for monitoring air pollution is to achieve a level of sensitivity to give quantitative insights into air quality with little cross-sensitivity, especially to water vapor. Table 2 outlines the *Ambient Air Quality Standards* set by the United States Environmental Protection Agency (EPA) for NO_2_, CO, and O_3_.^135^ The lower limit of quantification for any air pollution sensor needs to be in a range to make an assessment about the hourly air quality possible.

**Table 2:** Air Quality Standards According to the “Clean Air Act” Amended in 1990^135^

gas	averaging time	level
nitrogen dioxide (NO_2_)	1 h	0.100 ppm
	1 year	0.053 ppm
carbon monoxide (CO)	1 h	35 ppm
	8 h	9 ppm
ozone (O_3_)	8 h	0.070 ppm

In recent years, several carbon-based printed sensors for the detection of CO, NO_2_, and O_3_ have been reported.^100^ Early inkjet-printed carbon-based (graphene or CNT) chemiresistive sensors showed response from medium to high concentration of CO (0.1%) and NO_2_ (10 ppm).^167,168^ For NO_2_, the lower limit of detection for the inkjet-printed carbon-based sensors were 0.500 ppm for graphene-based^120,169^ and 0.250 ppm for CNT-based^119^ sensors. These values approach the limit of quantification required (0.100 ppm, Table 2), but are not sufficiently sensitive for quantitative monitoring of air quality.

A different technique, electrospray printing of graphene layers, can create a chemiresistive sensor capable of detecting NO_2_ (0.200 ppm) and O_3_ (0.050 ppm).^170^ The active sensing layer (reduced graphene oxide) is electro sprayed in a liquid suspension onto copper electrodes. The sensor proposed is capable of detecting NO_2_ and O_3_ at a relevant range but is highly sensitive to water vapor. The cross-sensitivity to water vapor is a major problem in atmospheric real-time gas measurements.

Inkjet printing of SnO_2_ (one of the most used metal oxides for gas sensing) on flexible and rigid substrates for sensing NO_2_ and CO, has been reported.^171−175^ The polyimide-based flexible sensor comprised gold electrodes, SnO_2_ sensing material, and gold heater, all of which were inkjet printed. This fully printed platform detected NO_2_ down to 0.600 ppm (with a calculated LOD of 0.001 ppm in dry air).^61^ An improved composition of SnO_2_ ink was able to detect CO down to levels of 5 ppm in dry air. Inkjet-printed CuO has also been used for measuring the air pollutant NO_2_.^176^ The sensor was produced by inkjet printing CuO on a silicon microheater. Through pulsed temperature modulation (100 °C/500 °C), the power consumption was decreased to 55 mW. The CuO-based sensor was able to detect 0.500 ppm of NO_2_ selectively in the presence of acetaldehyde and formaldehyde at 30% RH. As with all MOS sensors, sensitivity to water vapor is still an issue that needs addressing to enable real-world use.

Commercially, *SPEC Sensors*(112) offers printed electrochemical sensors for air quality monitoring. Their sensors appear to exhibit reasonable sensitivity to O_3_ (LOD = 0.028 ppm, calculated as 3× the standard deviation of the baseline), CO (<0.250 ppm, from raw data) and NO_2_ (0.012 ppm (calculated) and 0.100 ppm (measured)).^177,178^ The long-term stability (over 8 h), however, is ±0.150 ppm (for O_3_) which lies above the calculated LOD. This low stability, and the fact that no tests on the influence of changing levels of RH are available, neither from the datasheet nor from publications, makes it hard to estimate the usefulness of these sensors under real-world use cases. Additionally, the cost of one sensor package currently is $20^112^ which is not sufficiently low cost for many high-volume environmental applications.

Agricultural activity is a major contributor to environmental pollution, especially the overuse of soil additives to support growth of crops: Grell and co-workers developed a paper-based printed gas sensor to measure soil ammonium (NH_4_^+^) on a point-of-use basis.^179^ Their sensing system comprised a disposable cartridge that contained a cellulose paper-based substrate with printed carbon electrodes based on the work by Barandun et al.^55^ The soil solution (sample) was added to the cartridge and the pH was increased to 14 by adding sodium hydroxide to the sample. Increasing the pH shifts the equilibrium from NH_4_^+^ toward NH_3(aq)_ and eventually NH_3(g)_, which is detected by the printed gas sensor. To improve selectivity to NH_3_, the sensor was functionalized with sulfuric acid. Using this method, the authors demonstrated that a printed gas sensor can be used to measure soil ammonium levels to prevent overfertilization and its downstream environmental impact.

Quintero et al. showed a sensor system capable of detecting RH, ammonia and temperature, all printed and wirelessly accessible using Radio Frequency Identification (RFID) technology.^180^ An integrated RH sensor can reduce the cross-sensitivity of water vapor on the sensing of the target gas via pattern recognition or machine learning.

## Health

Noninvasive monitoring of health using printed gas sensors mainly focuses on exhaled breath, breathing patterns, and odors originating from the body.

### Exhaled Breath

Human breath is a complex mixture of gases, containing N_2_, O_2_, CO_2_, H_2_, H_2_O (main constituents), inorganic compounds (NO_2_, NO, NH_3_, CO, H_2_S), and up to 3500 VOCs.^181,182^ Clinical trials have shown that the levels and presence of volatile molecules in exhaled breath can be used in the diagnosis of asthma,^183^ diabetes,^184^ cancer,^185^ kidney disorders,^186^ and other conditions.^187−189^ Detecting individual biomarkers in this complex mixture is challenging and often requires expensive apparatus or a combination of sensors (sensor array, e-nose).^181,190^ There is a lack of low-cost, reliable, easy-to-use, diagnostic tools that can be used by minimally trained personnel. This is especially problematic for remote hospitals in low-resource settings.^191^ A low-cost and easy-to-use printed breathalyzer has to address the issues outlined below to be viable in a clinical environment for the detection of diseases.

i. *Humidity*: Exhaled breath contains large levels of water vapor which creates a highly humidified environment (>90% RH). A breathalyzer has to account for the variations in the levels of RH. Most low-cost sensors struggle with cross-sensitivity to water vapor.
ii. *LOD*: Optimally, the LOD needs to lie below the concentration of the biomarker of interest in the exhaled breath of healthy humans (for example, ∼1 ppm for ammonia).^192^ To detect raised levels of biomarkers in breath, the LOD should be well below the mean concentration in patients with health problems (for example, ∼5 ppm for ammonia in patients with renal failure).^192^
iii. *Real-time measurement*: There are two options to analyze human breath: breathing directly into a device (breathalyzer) which contains the sensor; or patients are asked to breathe into a sealable bag (for example, Tedlar bag). The breath sample collected in a bag can be processed (for example, dried, condensed) and analyzed later in a controlled environment. Direct breathing into a hand-held measurement device is more convenient and faster but is more challenging due to cross-sensitivity to water vapor.
iv. *Cross-sensitivity:* Breath contains over 3,500 VOCs. It is a tremendous challenge to create sensors that can detect each compound specifically. For the detection of individual biomarkers, the cross-sensitivity needs to be addressed either by improving the sensing material or postprocessing the data (for example, pattern recognition in a sensor array or principal component analysis (PCA)).

**Figure 4:**
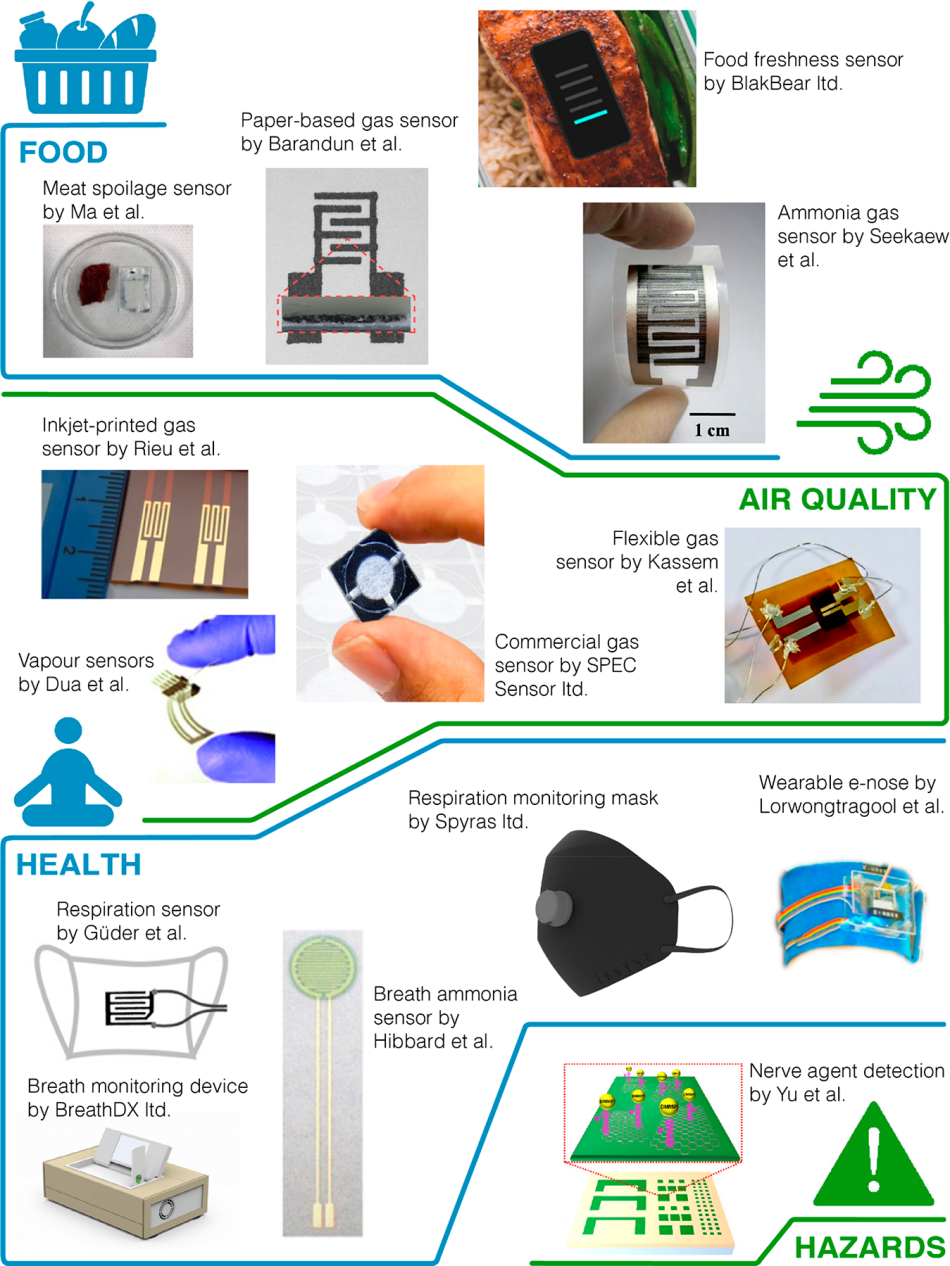
Printed gas sensors in different areas of application. **Panel food** (from left to right): Reprinted in part with permission from ref (154). Copyright 2018 American Chemical Society; Reprinted in part with permission from ref (55). Copyright 2019 American Chemical Society; Reprinted in part with permission from BlakBear Ltd. Copyright 2022 BlakBear; Reprinted with permission from ref (80). Copyright 2014 Elsevier. **Panel air quality**: Reprinted with permission from ref (175). Copyright 2016 Elsevier; Reprinted with permission from ref (120). Copyright 2010 John Wiley and Sons; Reprinted in part with permission from SPEC Sensors Ltd. Copyright 2022 SPEC Sensors; Reprinted with permission from ref (61). Copyright 2013 Royal Society of Chemistry; **Panel health**: Reprinted in part with permission from ref (201). Copyright 2016 John Wiley and Sons; Reprinted with permission from BreathDX Ltd. Copyright 2022 BreathDX; Reprinted with permission from ref (72). Copyright 2013 American Chemical Society; Reprinted with permission from Spyras Ltd. Copyright 2021 Spyras; Reprinted in part with permission from ref (197). Copyright 2014 MDPI; **Panel hazards**: Reprinted in part with permission from ref (204). Copyright 2019 American Chemical Society.

Ammonia is one of the most studied biomarkers in human breath and can indicate renal failure (for example, acute kidney injury).^131^ Hibbard et al. proposed a fully inkjet-printed ammonia sensor based on polyaniline nanoparticles on silver electrodes (Figure 4).^72^ With an LOD of 0.040 ppm, the sensor can detect concentrations of NH_3_ well below the mean of healthy levels (0.960 ppm). In a clinical test, their system was used to measure ammonia in pre- and postdialysis patients.^72^ The breath ammonia showed a correlation to blood urea nitrogen (BUN) with a Pearson coefficient *r* of 0.86–0.96 for 96 patients. The pre- and postdialysis measurements demonstrated a significant reduction of breath ammonia which correlated with BUN (*r* = 0.61, *p* < 0.01, *n* = 96). BUN is an indicator for renal function and is filtered out of the blood during dialysis. The disposable, printed ammonia sensors developed by Hibbard and co-workers are a promising alternative to monitor blood urea, hence measuring kidney health noninvasively. In 2016, a patent on their system was granted by the U.S. patent office followed by the European patent office in 2018.^193^ BreathDX is commercializing this technology with their AmBeR device.^115^

Maier et al. detected hydrogen peroxide (H_2_O_2_) in simulated breath with a system comprising a printed paper-based electrochemical sensor with a differential electrode design.^183^ The sensing (Prussian Blue mediated carbon), reference (silver/silver chloride), and counter (carbon) electrodes were all printed. Prussian Blue is a known electrocatalyst for H_2_O_2_ because it enables the detection of H_2_O_2_ at a potential near 0 V (vs Ag/AgCl). Their system detected H_2_O_2_ in real-time in simulated breath in a range of concentrations from 40 μM to 320 μM (approximately 1–10 ppm). The clinically relevant range of H_2_O_2_ in exhaled breath, however, is 2 orders of magnitude lower (0.005–0.050 ppm). The authors suggested that different PB content in the sensing electrode and modification procedures could help increase the sensitivity of their sensor to H_2_O_2_. Additionally, as with many real-time breathalyzers, their system is affected by the changing RH levels during inhalation and exhalation. The differential sensor design, however, helps eliminate the impact of water vapor on the measurement.

### Body Odor

Similar to human breath, human body odor contains a range of VOCs. Some of these VOCs are emitted from the axillary skin after being produced by metabolic processes^194^ and some originate from symbiotic bacteria living on the human skin.^195^ In contrast to human breath, less is known about body odor as an early indicator for disease. It is known, however, that human odor varies between individuals depending on genetics, diet, or levels of stress.^196^

Lorwongtragool et al. proposed a wearable e-nose for real-time tracking of body odors (Figure 4).^197^ The device can be worn as an armband and contains eight inkjet-printed sensors. Each individual sensor consists of interdigitated silver electrodes and a CNT-polymer composite for the sensing material. Upon exposure to gaseous analytes, the electrical resistance of each sensor changes to a different degree. Through mathematical modeling, a unique fingerprint can be created for each target gas. The authors of the study exposed the sensor array to 500 ppm of ammonia, acetic acid, acetone, and ethanol, which all showed a distinguishable pattern in a closed system. In a second experiment, the body odor of the armpits of three subjects were monitored before, during, and after exercise and yielded distinguishable patterns after PCA. The PCA clusters, however, vary between subjects and show some overlap between activities. Real-time body odor monitoring using an array of printed sensors (e-nose), although promising, requires further research to validate its utility for health monitoring or disease detection.^198^

### Breathing Pattern

Changes in breathing rate and volume can indicate a number of health issues including pulmonary disease, pneumonia, asthma, or cardiac arrest.^199^ The average human breathes between 10 and 20 times per minute and a change in breathing rate can be the result of cardiac arrest (higher breathing rate) or sleep apnea (paused breathing, hence lower breathing rate during sleep).^200^ Breathing patterns can easily be monitored in common medical settings, although expensive instruments are often required, making it inconvenient to continuously monitor the breathing rate over an extended period of time or in a nonstationary way. Güder et al. developed a printed, low-cost, humidity sensor implemented into a disposable facemask (Figure 4) that measured breathing rate by exploiting the difference in RH between inhaled and exhaled breath.^201^ The substrate, pure cellulose paper, is also the sensing material. Paper is hygroscopic and adsorbs moisture from its surrounding which changes its ionic conductivity. The sensor was probed by monitoring the resistance of paper using interdigitated carbon electrodes printed on the paper substrate. The low-cost ($0.005 for materials and $1.50 for the mask) and easy-to-use approach make this a technology an alternative to the current methods used for monitoring breathing. SPYRAS Ltd. has commercialized this technology.^111^ In the U.S.A., a patent around this cellulose-based sensor technology has been granted by the U.S. patent office in 2020.^202^

## Hazardous Gases

Chemical warfare agents (CWAs) and explosives are mostly color- and odorless, therefore, hardly detectable by humans. They can be lethal in low concentrations of parts-per-billion (ppb) down to parts-per-trillion (ppt).^203^ CWAs can be detected with sufficient sensitivity and selectivity by standard analytical methods (for example, gas chromatography or infrared spectroscopy). These methods, however, are difficult to perform in the field, require highly specialized personnel, do not allow real-time monitoring, and are expensive.

Yu et al. screen-printed a PANI/graphene composite onto cellulose paper to detect dimethyl methylphosphate (DMMP).^204^ Because of the lethality of many nerve agents, DMMP is often used as a replacement since it models the behavior of nerve gases without the high toxicity. The PANI/graphene composite functioned as a chemiresistive sensor and was able to detect DMMP down to 3 ppb. The cross-sensitivity with methanol, ethanol, ammonia, chloroform, and nitrogen dioxide at 300 ppb was between 0% and 30%. The approach reported had sufficient performance to be used as a low-cost sensor for detecting nerve agents suitable for mass production. The substrate (cellulose paper) had to be pretreated (coated) with a copolymer to improve the intermolecular forces between the paper substrate and PANI/graphene sensing material adding complexity to manufacturing.

Fang et al. reported a graphene oxide chemiresistive sensor inkjet-printed on a polyimide substrate.^205^ The sensor was able to clearly detect diethyl ethylphosphonate (DEEP), a nerve agent simulant, down to 2 ppm. The polyimide substrate required extensive pretreatment (that is, cleaning, surface modification, drying, and so forth) before printing, rendering manufacturing complex and potentially adding to the cost of the sensor. The detected concentration (2 ppm) is above the levels of interest (ppb) and the LOD was not determined in the study for the sensor produced.

Generally, the field of hazardous gas detection requires extremely sensitive devices. Compared to the limits in food spoilage, health, and air quality, where an LOD in the lower ppm range is acceptable, the LOD for CWAs lies much lower, in the range of ppb to ppt. Additionally, a device to detect CWAs needs to be highly reliable since a failed detection can have fatal consequences.

## Challenges and Future Opportunities

Printed electrical gas sensors show promising characteristics. Many of the printed gas sensing technologies, however, are not sufficiently mature yet and the following challenges need to be addressed in the future:

### Power Consumption

Recent advances in printed battery technologies allow many elements of batteries to be printed^206^ or integrated into the sensing substrate.^207^ Materials for the batteries are often difficult to recycle. Printed batteries also generally have lower energy densities than conventional batteries. Power consumption is, therefore, a major problem for sensors operated at elevated temperatures (mainly MOS sensors).^175^ Most other sensing technologies, such as electrochemical sensors, are low-power and can be powered passively. Passive power can be supplied by inductive coupling (NFC) or harvesting electromagnetic waves (UHF, Bluetooth low energy (BLE)). The power is provided by a reader (for example, a smartphone) and the sensing device is powered passively by an antenna which can simultaneously be used for data transmission. Combinations of printed sensors and passive power technologies have been demonstrated.^55,154,167^ The antenna can potentially be printed with the same conductive material used for the sensing electrodes, eliminating additional manufacturing steps. A shortcoming of a passively powered device is that the reader needs to be nearby—a few centimeters for NFC and a few meters for UHF and BLE—to provide power. The readers, however, can supply power and communicate wirelessly to a high number of sensors simultaneously with anticollision protocols.

### Sensor Performance

Common approaches to increase sensitivity and/or selectivity^208^ (for example, temperature controlled operation modes, preconcentration of the target gas, or separation of the analytes (gas chromatograph)) are not viable for printed, low-cost sensors. These approaches increase the complexity and cost of the system, often requiring additional components which cannot be printed. Viable options to increase the performance of printed gas sensors are as follows: (i) filters (for example, membranes) selectively filter the analyte before the gas mixture is in contact with the sensing element; (ii) machine-learning recognizes a distinct pattern generated by the analyte in a mixture of gases; (iii) combination of different sensors into a sensor array (e-nose) to create a unique fingerprint of the analyte. In an e-nose, sensors (generally >3) are combined into an array and a pattern recognition system assigns the combined response generated by the sensors to an analyte. The idea of an e-nose has been around for over 50 years,^209^ but commercial options are just emerging^210^ with no simple and low-cost solutions being available yet. Because of their ease of production and low cost, printed sensors are a great potential candidate to be combined into arrays to build the next generation of e-noses.^180^

### Membranes

Membranes can increase selectivity by filtering for the target gas and stop water (liquid and vapor) and other contaminates to reach the sensor, which would deteriorate sensing performance. Most printed gas sensors reported work in a controlled (clean) lab environment and, therefore, do not require a protective membrane. For future fully printed gas sensing devices, the printing of the protective membrane is a requirement. A common membrane material is polytetrafluoroethylene (PTFE) or derivatives thereof which are highly hydrophobic (water repellent). 3M recently started to provide 3D printing options for PTFE parts.^211^ There are also reports of inkjet-printed PTFE.^212^ It is, however, not yet possible to print a thin enough layer of PTFE (a few 100 μm) to act as a protective, gas-permeable membrane.

### Disposal

For a device to be truly disposable, not only does the cost need to be near zero, but the contained materials should be recyclable, environmentally friendly or, at least, nontoxic.^213^ Commonly used recyclable materials in printed sensors include natural materials (cellulose paper), metals (copper, aluminum), or silicon. Sensing platforms include, inks, electronics, metals, substrates, membranes, and are often a combination of embedded materials that are difficult to recycle. The recyclability of these systems presents a major challenge for their future integration into recycle products.

**Figure 5:**
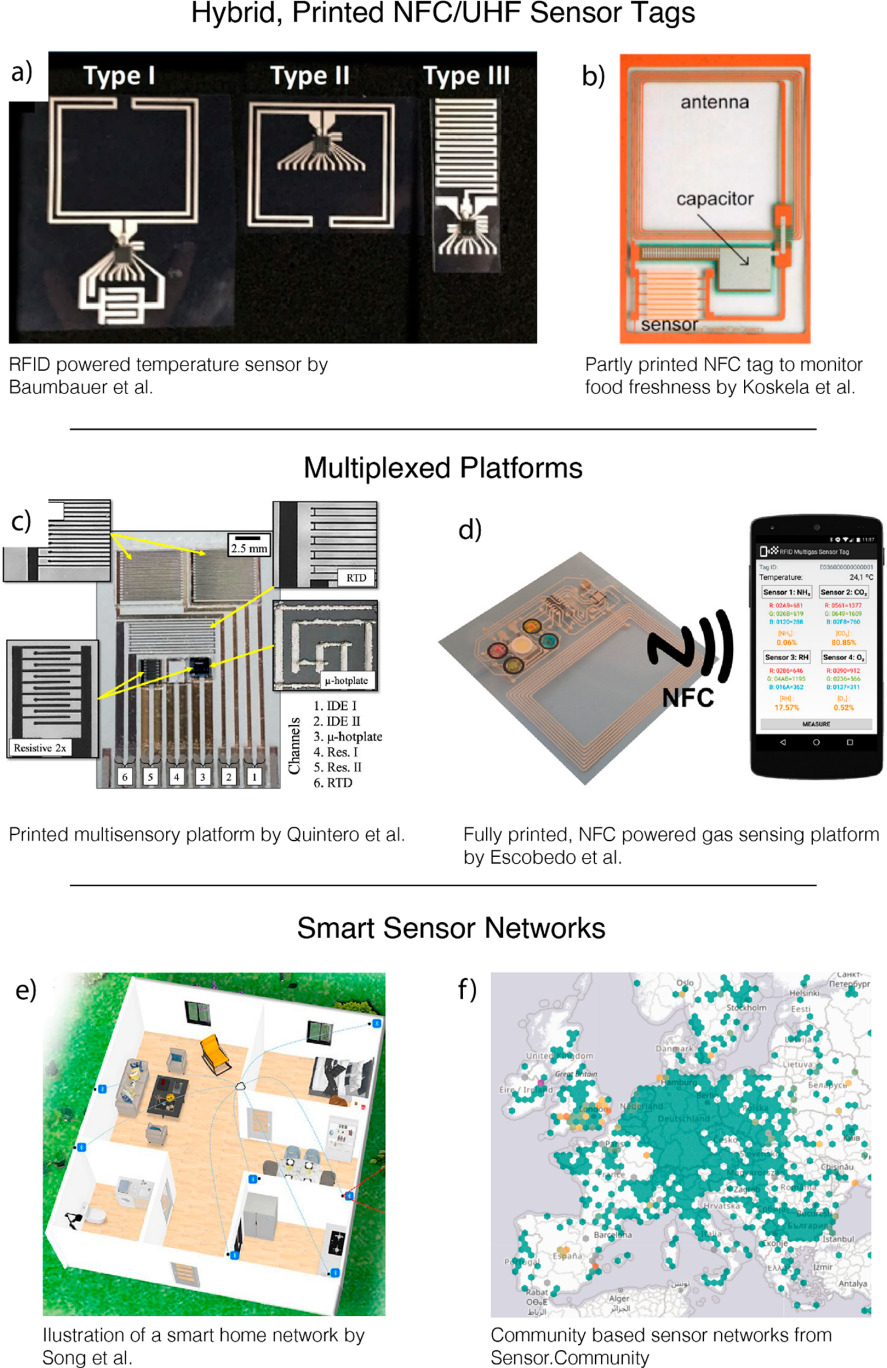
Future applications of printed gas sensors. (a) RFID powered temperature sensor by Baumbauer et al. Reprinted with permission from ref (214). Copyright 2020 Springer Nature; (b) Partly printed (antenna not printed) NFC tag to monitor the freshness of poultry by Koskela et al. Reprinted with permission from ref (145). Copyright 2015 Elsevier; (c) All printed multisensory platform by Quintero et al. Reprinted with permission from ref (180). Copyright 2016 Institute of Physics Publishing Ltd.; (d) Fully printed, NFC powered gas sensing platform by Escobedo et al. Reprinted with permission from ref (86). Copyright 2016 American Chemical Society; (e) Smart homes by Song et al. Reprinted in part with permission from ref (215). Copyright 2021 American Chemical Society; (f) Community based sensor networks from https://sensor.community/en/.

An opportunity that stems from the low power consumption of printed gas sensors is the possibility of battery-free operation using WPT. The antennas and coils used for WPT can be printed, allowing seamless integration into fully printed devices.^85^ Baumbauer et al. demonstrated different options to create a flexible, hybrid UHF tag (inkjet printing, spray coating, screen printing, and pencil coating).^214^ The only nonprinted part was the silicon chip for RFID communication (Figure 5). They did not apply the tag for gas sensing measurements, but their approach can easily be combined with low-power printed gas sensing elements to create hybrid RFID tags.

A wirelessly powered, disposable printed electrical gas sensor meets many of the requirements for smart homes, including small formfactor, low cost and ultralow power consumption.^215^ Gas sensors for smart homes monitor air quality (mainly CO_2_ and O_2_) and detect hazardous gases (H_2_ and CO). A system reported by Song et al., can be the basis for a future fully printed gas sensor network to monitor air quality and hazardous gases in homes (Figure 5).^215^ The advantage of a higher resolution of sensors includes the possibility to determine the location of leakages instead of only detecting the presence of hazardous gases.^215^

Sensor arrays can be a powerful tool as demonstrated by Raskow and Suslick.^216^ All-printed multianalyte platforms based on WPT are the most promising perspectives for printed electrical gas sensors. The possibilities of these systems have been shown in parts in the past (Figure 5).^86,217,218^ The printing technologies allow full printing of said devices including, sensing element, communication (antenna), and power unit (antenna, coil). The only rigid parts left are generally electronic chips used for amplification or communication (RFID chip). These platforms can already sense RH and multiple gases, such as CO, CO_2_, NO_2_, O_2_, and NH_3_. Printing sensors allows for fast and low-cost assembly of a variety of different sensors or identical sensors with different functionalization to be combined into a sensor array. The array needs to be trained on the gases of interest to create a databank of responses under different conditions and in different gas mixtures, inspired by the human olfactory system.

## Conclusions

Printed gas sensors are expected to fill a large gap in the current technological landscape for low-cost, low-power, and high-performance analytical systems to democratize gas sensing.

Printed gas sensors offer at least three major advantages in comparison to other gas sensing technologies: (i) Printed gas sensors can be prototyped and manufactured at scale with commonly available instruments and techniques such as screen printing. (ii) Because printed gas sensors can be disposable, low-power, flexible, stretchable, and small, they can be placed in locations that would not be suitable for other gas sensing technologies, for example, curved surfaces and low-cost packaging. (iii) Because most printed gas sensors are electrical, they can be easily integrated into Internet-of-Things (IoT) digital networks to connect chemical and biological systems with machines and networks of machines (Figure 5).^219^ These specifications allow for accessible and affordable sensing units which opens many opportunities, for example, the creation of community based environmental monitoring networks (Figure 5). Similar platforms already exist with conventional sensing technology at much higher cost (>$50 per device).^220^ Printed electrical gas sensors can also be a part of smart-contract-based, trustless, blockchain networks, such as Ethereum, to automate various processes and transactions in a distributed fashion.^221,222^

Printed gas sensors will require continued improvements with respect to sensitivity, selectivity, and analytical robustness to replace more expensive, conventional technologies for applications that demand high sensitivity, selectivity and reliability. According to the forecasts in 2017, the market for “fully printed sensors” was predicted to be $7.6 billion in 2027.^223^ Even though the market size was recently revised to $4.9 billion for 2032, it is clear that printed sensing technologies, including gas sensors, have a significant potential.^224^ As the challenges concerning printed electrical gas sensors are addressed, the true potential of this emerging technology will be realized, especially in applications concerning healthcare, food, and environmental monitoring.
